# Ocean acidification modulates expression of genes and physiological performance of a marine diatom

**DOI:** 10.1371/journal.pone.0170970

**Published:** 2017-02-13

**Authors:** Yahe Li, Shufang Zhuang, Yaping Wu, Honglin Ren, Fangyi Chen, Xin Lin, Kejian Wang, John Beardall, Kunshan Gao

**Affiliations:** 1 State Key Laboratory of Marine Environmental Science (Xiamen University), College of Ocean and Earth Sciences, Xiamen University, Xiamen, China; 2 Key Laboratory of Zoonosis Research, Ministry of Education, Institute of Zoonosis, Jilin University, Changchun, China; 3 School of Biological Sciences, Monash University, Clayton, Victoria, Australia; Stazione Zoologica Anton Dohrn, ITALY

## Abstract

Ocean Acidification (OA) is known to affect various aspects of physiological performances of diatoms, but little is known about the underlining molecular mechanisms involved. Here, we show that in the model diatom *Phaeodactylum tricornutum*, the expression of key genes associated with photosynthetic light harvesting as well as those encoding Rubisco, carbonic anhydrase, NADH dehydrogenase and nitrite reductase, are modulated by OA (1000 μatm, pH_nbs_ 7.83). Growth and photosynthetic carbon fixation were enhanced by elevated CO_2_. OA treatment decreased the expression of β-carbonic anhydrase (*β-ca*), which functions in balancing intracellular carbonate chemistry and the CO_2_ concentrating mechanism (CCM). The expression of the genes encoding fucoxanthin chlorophyll *a*/*c* protein (lhcf type (*fcp*)), mitochondrial ATP synthase (*mtATP*), ribulose-1, 5-bisphosphate carboxylase/oxygenase large subunit gene (*rbcl*) and NADH dehydrogenase subunit 2 (*ndh*2), were down-regulated during the first four days (< 8 generations) after the cells were transferred from LC (cells grown under ambient air condition; 390 μatm; pH_nbs_ 8.19) to OA conditions, with no significant difference between LC and HC treatments with the time elapsed. The expression of nitrite reductase (*nir*) was up-regulated by the OA treatment. Additionally, the genes for these proteins (NiR, FCP, mtATP synthase, β-CA) showed diel expression patterns. It appeared that the enhanced photosynthetic and growth rates under OA could be attributed to stimulated nitrogen assimilation, increased CO_2_ availability or saved energy from down-regulation of the CCM and consequently lowered cost of protein synthesis versus that of non-nitrogenous cell components.

## Introduction

Ocean acidification (OA), expressed in milieu as a decline in pH, is driven by rapid increases in CO_2_ taken up by the oceans from the atmosphere and is altering marine chemical environments with consequences for marine organisms and the biological CO_2_ pump [1]. Although intracellular pH levels of both photosynthetic organisms and animals are known to be below that of the bulk seawater pH [2], external pH decline is known to affect the physiology of many marine organisms to different extents [3]. For instance extracellular pH changes can influence the membrane electrochemical potential and enzyme activity [4–6]. Additionally, different and controversial responses of diatoms to OA have been reported [7]. For instance, in the model diatom *Phaeodactylum tricornutum*, growth and photosynthetic carbon fixation rate were enhanced when cells were acclimated to 1000 μatm CO_2_ under indoor low light conditions [8–10], but became inhibited under elevated pCO_2_ levels under fluctuating high sunlight levels [9]. The active CO_2_ acquisition process, the CO_2_ concentrating mechanism (CCM), is known to be down-regulated under increased CO_2_ levels [11], which may increase light stress under high light levels but conversely can enhance the growth of diatoms under low light levels [9]. Consequently, changes in the energy budget of diatoms grown under OA conditions could alter the energy costs for protein synthesis versus that of non-nitrogenous cell components [12, 13]. Photophysiological performance could also be altered, since some microalgae that lack NDH-1 require a plastidial NDH-2 in cyclic electron flow (CEF) to produce extra ATP needed for CO_2_ fixation [14, 15].

In addition to the physiological responses to OA, molecular responses indicate that the expression of α- and γ-CA genes, in *Emiliania huxleyi*, was down-regulated by elevated CO_2_ [16] as was the δ-CA in *Thalassiosira pseudonana* [17, 18]. In *P*. *tricornutum*, the two chloroplastic β-CAs, PtCA1 and 2, have previously been shown to be CO_2_ responsive [19–22] and mediated by light levels [20]. On the other hand, the evidence for a role of the urea cycle in linking N and C metabolism, reported in diatoms [23, 24], is still controversial. Therefore, from both physiological and molecular points of view, there is a need to further comprehend the mechanisms involved in the responses of marine organisms to OA.

Responses of diatoms to elevated *p*CO_2_ or OA must be considered in the context of other ocean changes, such as ocean warming, increased exposure to solar radiation and reduced nutrient availability due to warming-enhanced stratification [17, 25–27]. In the present study, we chose *P*. *tricornutum*, whose genome has been completely sequenced [28], as the material to examine the relationship between physiological performance and genetic responses. Considering the associated key gene products and the debate concerning the role of the urea cycle, the expression of the fucoxanthin chlorophyll *a*/*c* protein (lhcf type (*fcp*)), ribulose-1, 5-bisphosphate carboxylase/oxygenase large subunit gene (*rbcl*), β-carbonic anhydrase (*β-ca*), mitochondrial ATP synthase (*mtATP*), NADH dehydrogenase subunit 2 (*ndh*2), peroxisomal membrane protein (*pmp*) and nitrite reductase (*nir*) were investigated after the diatom was acclimated to the projected levels of future ocean acidification under different light treatments.

## Materials and methods

### Species and culture conditions

The strain of the diatom *Phaeodactylum tricornutum* Bohlin (strain CCMA 106), originally isolated from the South China Sea in 2004, was obtained from the Center for Collections of Marine Bacteria and Phytoplankton (CCMBP) of the State Key Laboratory of Marine Environmental Sciences (Xiamen University). No specific permits were required for the using of this species. Although this is not the strain (CCMP632) used for sequencing of the *Phaeodactylum* genome [28], we used it because of its recent isolation and regional importance. The unialgal culture of this diatom was maintained in filtered (0.22 μm) and sterilized seawater collected from the South China Sea (18° N, 116° E), enriched with Aquil medium [29], under 130 μmol m^-2^ s^-1^ of Photosynthetically Active Radiation (PAR, L: D = 12: 12, with the light period beginning at 8:00 am) and 20°C before the experiment. During the experimental periods, the cultures were aerated with ambient (at the time of the experiment) air (390 μatm, LC; from the roof of the building) or elevated CO_2_ (1000 μatm; HC) in air, in an illuminated CO_2_ plant incubator (HP1000G-D, Wuhan Ruihua Instrument & Equipment Ltd, China) under the same light and temperature conditions. The HC was achieved automatically within the CO_2_ plant chamber with less than 4% variation in the CO_2_ concentration. Cells were grown as semi-continuous cultures and between the dilutions (with dilution every 24 h), cell concentrations were maintained within a range of 7×10^4^–2.8×10^5^ cell mL^-1^ to maintain stable seawater carbonate chemistry (S1 Table).

### Experimental set up

In order to evaluate the effects of elevated CO_2_ (1000 μatm; HC) during both light and dark periods and/or different times of day, sampling for both physiological and genetic measurements was carried out at various times during the light/dark cycle after cells were transferred from LC to HC conditions. Sampling times (with light/dark conditions indicated as l or d respectively) were set as: 4 h-l, 8 h-l, 16 h-d, 28 h-l, 32 h-l, 40 h-d, 76 h-l, 80 h-l, 88 h-d, 172 h-l, 176 h-l, 184 h-d (the concept map for this design is shown in S1 Fig). Following the internationally recognized OA research guide [30], 10 generations were considered sufficient for cells to become acclimated to LC or HC levels.

### Growth rates

At the end of the light period, dilution was performed with one part of the culture added to two parts autoclaved seawater, equivalent to a dilution rate of 0.67 d^-1^ (the seawater was enriched with Aquil medium and pre-equilibrated with the targeted CO_2_ levels). The cell densities in the semi-continuous cultures were counted immediately before and after the dilution (every 24 h) using a particle counter (Z2, Beckman, USA). The specific growth rate (μ, d^-1^) was calculated as: μ = (ln C_1_−ln C_0_) / (t_1_−t_0_), where C_0_ is the initial (after dilution) cell concentration and C_1_ that (before the next dilution) after 24 h.

### Chlorophyll fluorescence parameters

The maximal photochemical efficiency, F_v_/F_m_ was measured after 15 minutes dark adaptation using a Xenon-Pulse Amplitude Modulated fluorometer (XE-PAM, Walz, Germany). The relative electron transport rate (rETR, arbitrary units) was calculated as: rETR = F_v_′/F_m_′×0.5×PAR, where F_v_'/F_m_' represents the effective PSII quantum yield, PAR is the photosynthetically active photon flux density (μmol m^-2^ s^-1^) and the coefficient 0.5 takes into account that approximately 50% of all absorbed photons reach PSII. The rapid light curves (RLCs) were measured under eight different PAR levels (every measurement lasted for 10 s). RLCs were fitted as rETR = E/(aE^2^+bE+c) [31], where E is PAR (μmol m^-2^ s^-1^) and a, b and c are derived parameters. The maximum relative electron transport rate (rETR_max_) was expressed as a function of the parameters a, b, and c as follows: rETR_max_ = 1/(b+2(a×c)^1/2^). Non-photochemical quenching (NPQ) was calculated as: NPQ = (F_m_-F_m_')/F_m_', where F_m_ was the maximum fluorescence yield after dark adaptation and the F_m_', the maximum fluorescence yield under the actinic (growth) light levels.

### Determination of photosynthetic carbon fixation rate as a function of DIC concentration

After cells had been acclimated to LC and HC for 10 generations, the relationship of photosynthetic carbon fixation rate with external dissolved organic carbon (DIC) concentration in seawater (P-C curve) was determined at 20°C and 400 μmol m^-2^ s^-1^ using ^14^C-labeled sodium bicarbonate (Amersham) as described previously [32].

The cells were harvested during the mid-photoperiod by filtering onto hybrid fiber membrane (1μm, Xinya, Shanghai, China), then washing and re-suspending in DIC-free seawater (pre-buffered with 20 mmol L^-1^ Tris-HCl at pH 8.18) at a final concentration of about 2×10^5^ cells mL^-1^. The DIC-free seawater was prepared according to Gao *et al*. [33]. The DIC concentrations (50–3200 μmol L^-1^) of the medium were adjusted by adding NaHCO_3_ solution into cell suspensions prepared with DIC-free seawater. The maximal carbon fixation rate and the K_1/2_ values for DIC were determined by fitting the rates of photosynthetic carbon fixation at various DIC concentrations to the Michaelis-Menten equation [34].

### Determination of gene expression

Expression levels of the targeted genes were validated by quantitative reverse transcriptase-polymerase chain reaction (q-RT–PCR), which was performed in a 7500 real-time PCR system (Applied Biosystems). Total RNAs were extracted using RNeasy@ Plant Mini Kit following the manufacturer’s instructions (QIAGEN) and quantified with a NanoDrop 2000 microvolume spectrophotometer (Thermo Scientific). One microgram of total RNAs for each group was separately reverse-transcribed in a final volume of 20 μL using a PrimeScript^TM^ RT reagent kit (Perfect Real Time) (TaKaRa) following the manufacturer's instructions. Real-time PCR was performed in a reaction mixture of total transcribed cDNA, gene-specific primer and Power SYBR Green PCR Master Mix (Applied Biosystems, UK). The primers for β-carbonic anhydrase (*β-ca*; ID:Phatr2_51305), fucoxanthin chlorophyll *a*/*c* protein, lhcf type (*fcp*, annotated as *lhcf 3* in the *P*. *tricornutum* JGI database; ID: Phatr2_50705), ribulose-1, 5-bisphosphate carboxylase/oxygenase large subunit gene (*rbcl*; Chloroplast gene) mitochondrial ATP synthase (*mtATP*; ID: Phatr2_14618), peroxisomal membrane protein (*pmp*; ID:phatr2_22819), nitrite reductase (*nir*; not found in JGI) and NADH dehydrogenase subunit 2 (*ndh2*; Mitochondrial gene) are shown in S2 Table. The standard cycling conditions were 95°C for 10 min, followed by 40 cycles of 95°C for 15 s, 60°C for 25 s, and 72°C for 40 s. Raw relative data quantification was carried out using the 7500 system SDS software version 1.3.1.21, and the housekeeping histone H4 gene was employed as the internal standard [35]. The first sampling point in the Low CO_2_ group (LC group) was used as the calibrator.

### Data analysis

Three independent replicate cultures for each CO_2_ condition were used in all experiments, and the data are plotted as mean ± SD (standard deviation). Two sample t-tests were used to establish differences among the LC and HC treatments at each sampling time point with significance tests being done using a 95% confidence level.

## Results

### Growth and photosynthetic performance

**Fig 1:**
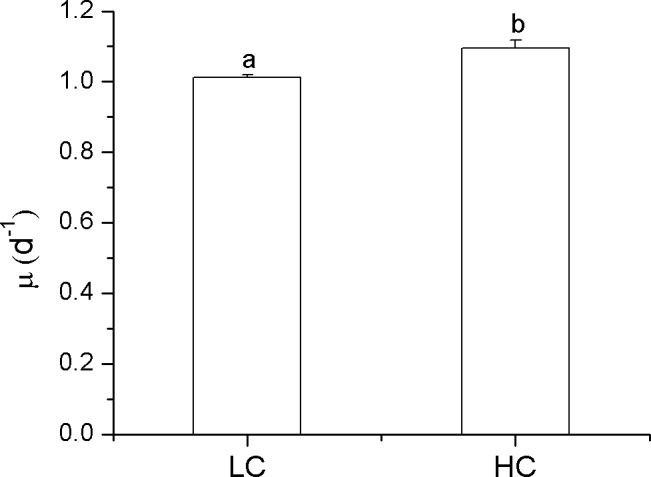
Specific growth rates of *P*. *tricornutum*. Specific growth rate (μ) of *P*. *tricornutum* cells grown at ambient (390 μatm; LC) and elevated CO_2_ (1000 μatm; HC) levels. Data are the means ± SD, n = 3 (triplicate cultures). Different letters above the histogram bars indicate significant differences (p<0.05) between different treatments.

**Fig 2:**
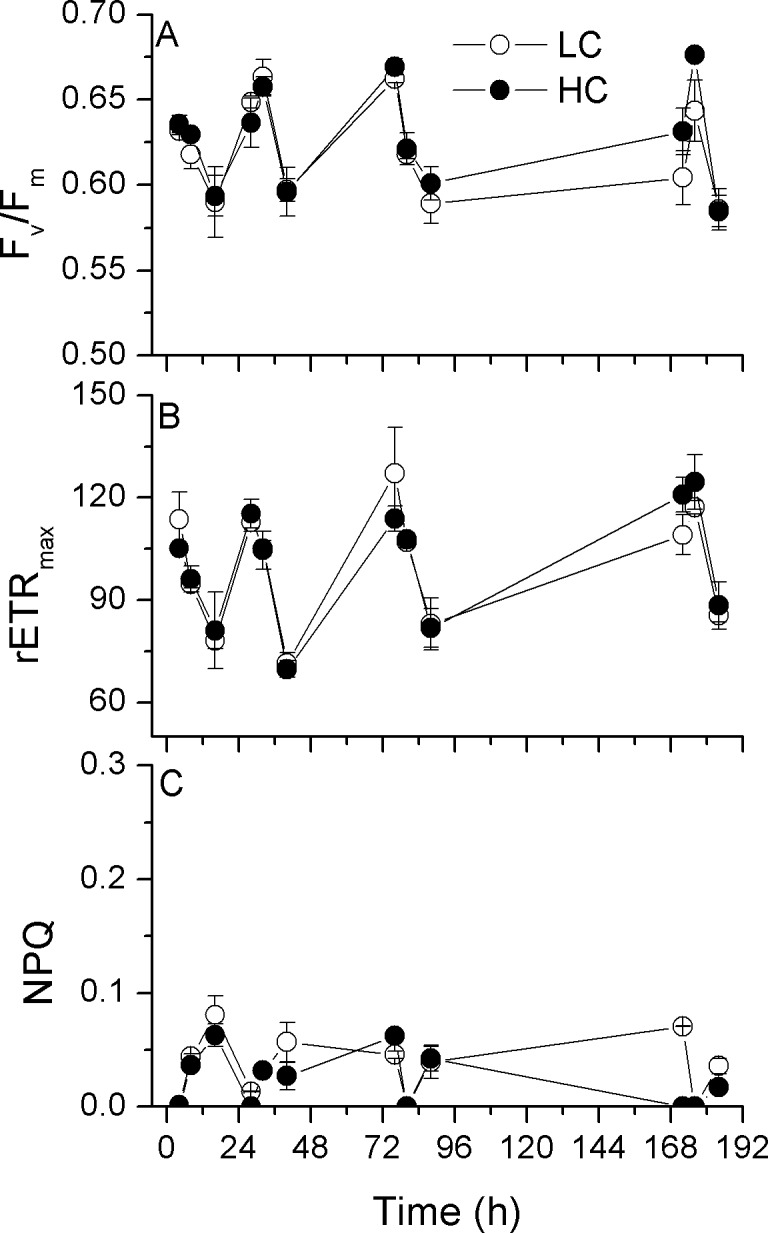
Chlorophyll fluorescence parameters of *P*. *tricornutum*. Time series of the maximal quantum yield (F_v_/F_m_; A), the maximal electron transport rate (rETR_max_; B) and non-photochemical quenching (NPQ; C) of *P*. *tricornutum* cells grown at ambient (390 μatm; LC) and elevated CO_2_ (1000 μatm; HC) levels. Data are the means ± SD, n = 3 (triplicate cultures).

The specific growth rate (μ; obtained from day 3 to day 8) of the diatom *P*. *tricornutum* was slightly enhanced by the elevated CO_2_ (*p* = 0.004) (Fig 1). The maximal photochemical yield of PSII, F_v_/F_m_, showed insignificant (*p* = 0.145) differences between the LC and HC-grown cells (Fig 2A). The maximal electron transport rate (rETR_max_) showed marked diurnal oscillations under all conditions (Fig 2B) and, after acclimation to the elevated CO_2_ concentration for ~8 generations, the HC cells showed slightly higher (by 3–11%) rETR_max_ (*p* = 0.03) compared to the LC-grown cultures. Additionally, the cells, no matter whether they were grown in LC or HC levels, showed low values of non-photochemical quenching (NPQ) (Fig 2C).

### CCM activity

**Fig 3:**
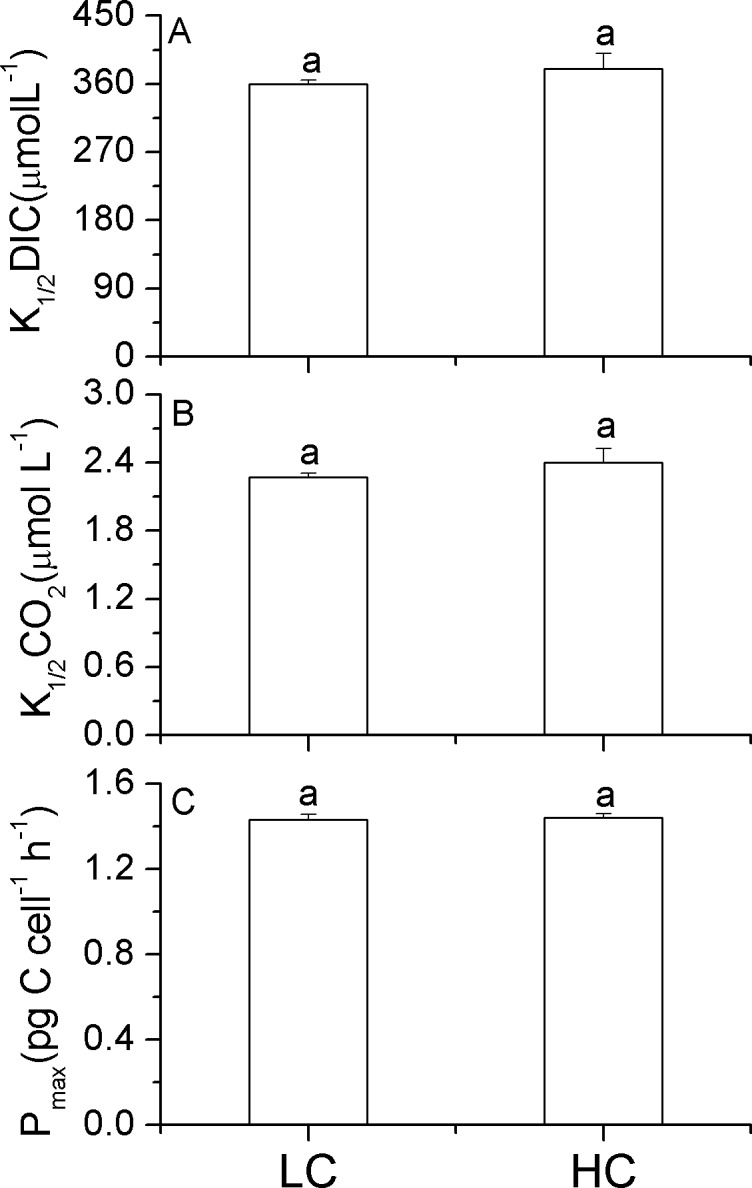
Half-saturation constants (K_1/2_) for DIC (A) and CO_2_ (B) and the maximal photosynthetic rate (C) of *P*. *tricornutum*. The half-saturation constants for dissolved inorganic carbon (A: K_1/2_ DIC; μmol L^-1^) or CO_2_ (B: K_1/2_ CO_2_; μmol L^-1^) concentrations and the maximal photosynthetic rate (C: P_max_; pg C cell^-1^ h^-1^) of *P*. *tricornutum* cells grown at ambient (390 μatm; LC) and elevated CO_2_ (1000 μatm; HC) levels. Data are the means ± SD, n = 3 (triplicate cultures). Different letters above the histogram bars indicate significant differences (p<0.05) between different treatments.

The HC-grown cells did not show significantly higher values of K_1/2_DIC (*p* = 0.16; Fig 3A), K_1/2_CO_2_ (*p* = 0.17; Fig 3B) or maximal photosynthetic rate (P_max_; *p* = 0.65 Fig 3C), compared to the LC-grown ones.

### Gene expression

**Fig 4:**
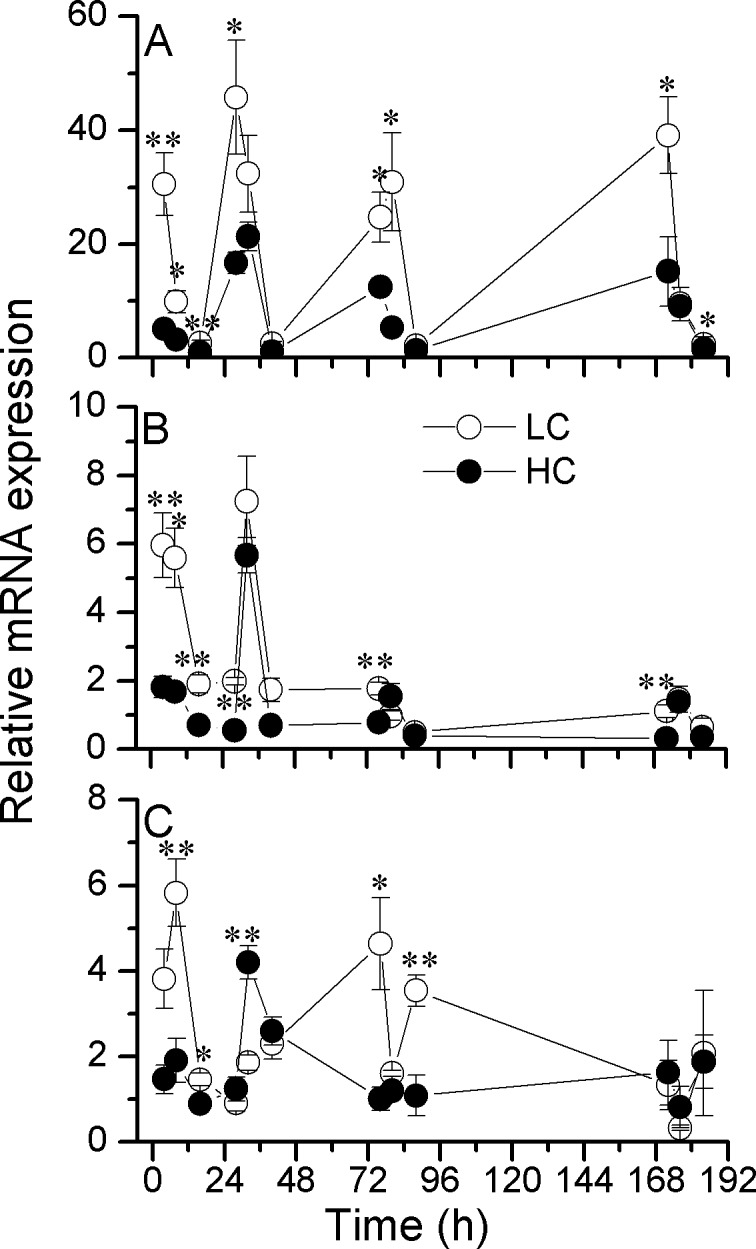
The relative abundances of transcripts for photosynthetic relevant genes. Time series of the relative abundances of transcripts for β-carbonic anhydrase (β-CA; A), fucoxanthin chlorophyll *a*/*c* protein, lhcf type (FCP, Lhcf 3; B), ribulose-1, 5-bisphosphate carboxylase/oxygenase large subunit gene (RbcL; C) determined by quantitative real-time PCR (qPCR) of *P*. *tricornutum* cells grown at ambient (390 μatm; LC) and elevated CO_2_ (1000 μatm; HC) levels. Data are presented as means ± SD, n = 3 (triplicate cultures). Two asterisks indicate a significant difference between HC and LC grown cells at *p* < 0.01, and one asterisk represents a significant difference at *p* < 0.05.

**Fig 5:**
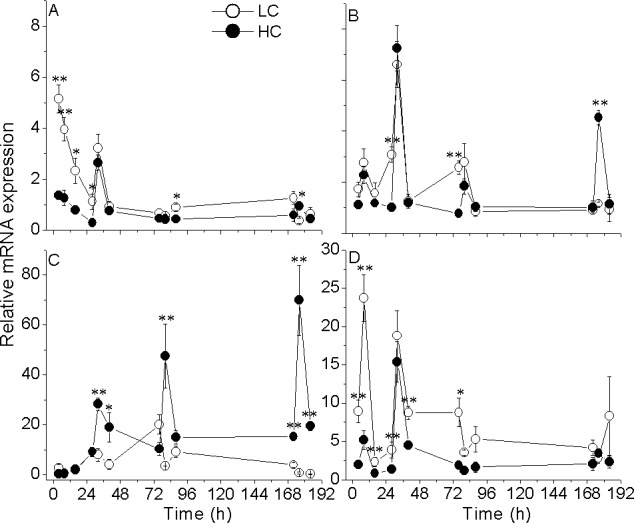
The relative abundances of transcripts for non-photosynthetic relevant genes. The time series of the relative abundances of transcripts for mitochondrial ATP synthase (mATP; A), peroxisomal membrane protein (PMP; B), nitrite reductase (NiR; C) and NADH dehydrogenase subunit 2 (Ndh2; D) determined by quantitative real-time PCR (qPCR) of *P*. *tricornutum* cells grown at ambient (390 μatm; LC) and elevated CO_2_ (1000 μatm; HC) levels. Data are presented as means ± SD, n = 3 (triplicate cultures). Two asterisks indicate significant difference between HC and LC grown cells at *p* < 0.01, and one asterisk represents a difference at *p* < 0.05

The expression levels of targeted genes showed obvious diel changes in both LC and HC-grown cells, especially for β-carbonic anhydrase (*β-ca*) (Figs 4 and 5). The expression of the *β-ca* gene decreased in the HC-grown cells (by up to 80%; *p* = 0.005), with the minima observed during the dark period (Fig 4A). The gene expression for *lhcf* 3, encoding the major antennae in the light harvesting complex (LHC), was significantly down-regulated (*p* = 0.04) after the cells had been exposed to HC for a period of 4–48 h, with the highest values obtained 8 h after the onset of light and the lowest values 4 h after the onset of darkness. Significantly decreased expression of *lhcf* 3 was observed as time elapsed (Fig 4B). For the *rbcl* gene, both down and up-regulation were observed in the HC-grown cells, although with increased time of acclimation to HC, there were no significant difference between the LC and HC treatments (Fig 4C). The expression of the mitochondrial ATP synthase (mtATP synthase) gene following cells’ transfer to HC decreased significantly (*p* = 0.03) at most of the measurement times except for that at 176 h (Fig 5A). For the peroxisomal membrane protein (PMP) gene, slight diel oscillations and both down- or up-regulated expression by elevated CO_2_ were observed (Fig 5B). However, the expression of the nitrite reductase (NiR) gene was significantly up-regulated by elevated CO_2_ (*p* = 0.03) (Fig 5C), while an inverse trend was observed in the gene encoding NADH dehydrogenase subunit 2 (NDH2), with the highest down-regulation by about 78% under the OA conditions (Fig 5D).

## Discussion

Phytoplankton cells within the upper mixed layers of the oceans are exposed to both increasing pCO_2_ and higher solar radiation due to enhanced thermal stratification. Interactions of these two key factors are crucial for predictions of the biological consequences of global change in the oceans. Here, we showed for the first time that the marine diatom *P*. *tricornutum* under elevated CO_2_ up-regulated a gene related to nitrogen assimilation while it down-regulated the gene for β-CA, a protein functioning in maintaining CO_2_ bicarbonate equilibrium, CO_2_ diffusion to Rubisco and CCMs within cells.

Changes in growth rates are supposed to be associated with altered gene expression [36]. The effect of OA on growth has been shown to be mediated by light levels [9] in cells that had acclimated to elevated CO_2_ over 20 generations. In previous work, this diatom showed enhanced growth rate under PAR of 50–150 μmol m^-2^s^-1^, and inhibited growth rates under daytime mean solar PAR >220 μmol m^-2^s^-1^, after the cells had acclimated to OA over 10 generations [8–10]. In the present work, growth of *P*. *tricornutum* was significantly enhanced by elevated CO_2_, which could be attributed to the high energy cost of protein synthesis versus that of non-nitrogenous cell components [12, 13], as well as energy savings from down-regulation of CCMs [8, 11]. In this study, the expression of *β-ca* was significantly down-regulated under the high CO_2_ condition, a phenomenon also observed in previous investigations [19, 20], though the accompanying increase in K_1/2_DIC and K_1/2_CO_2_ values were not significantly different between the ambient or elevated CO_2_ levels (Fig 3), while they were significantly different in our previous studies [8, 12]. Such an inconsistency might be attributed to the different growth light levels used, which are known to modulate the efficiency of CCMs [37, 38], or to the different number of generations for which the cells were acclimated to different CO_2_ levels. Additionally, the expression of *ndh2* was significantly down-regulated by elevated CO_2_, indicating possible down-regulation of CEF activity and lower requirements for extra ATP under the OA treatment [14, 15].

The expression of *nir* was significantly up-regulated by elevated CO_2_, suggesting that rate of N assimilation might have increased. The cellular PON did increase in *P*. *tricornutum* grown under the elevated CO_2_, as reported in another study [12], as well as in *Skeletonema costatum* [39], *E*. *huxleyi* [40], *Gephyrocapsa oceanica* [41] and *Coccolithus pelagicus* [42], though POC/PON ratios changed in a species-specific fashion under OA [26]. Although Shi *et al*. [43] showed down-regulated protein levels (but without significant changes to gene expression) and activity of nitrate reductase in *T*. *pseudonana* under high CO_2_ conditions regardless of light levels, and both nitrate reductase and nitrite reductase are components of the N assimilation pathway, they are different genes and may show different responses to changes in pH. Additionally, there are a number of genomic differences between *T*. *pseudonan*a and *P*. *tricornutum* [28, 44] and differential physiological responses to environmental changes have also been reported [7]. Therefore, species-specific responses and/or culture conditions could be responsible for the difference between the results of Shi *et al*. [43] and this paper. Moreover, Hofmann *et al*. [45] reported that the activity of nitrate reductase was stimulated by elevated CO_2_ in the calcifying rhodophyte *Corallina officinalis*. Chauton *et al*. [46] studied carbon fixation, storage and utilization in *P*. *tricornutum* acclimated to light/dark cycles and found that genes related to nitrogen metabolism were up-regulated when the cells were shifted from light to dark, which might be related to the pH drop during the dark period as cells respire and produce CO_2_. In the present study, however, the expression of the *nir* gene showed maximal expression during the middle of the light period and the diel expression pattern was amplified by elevated CO_2_ (Fig 5C). Obviously, the OA treatment stimulated expression of this gene. It appears that metabolic pathways involving nitrate uptake or nitrogen assimilation respond to OA for the cells to maintain homeostasis while suffering from acidification from the milieu. Additionally, although the urea cycle may be integrated into nitrogen metabolism through its connection to glutamine and in the eventual production of urea in *P*. *tricornutum* [23] and *T*. *pseudonana* [47], and there are some claims about the benefits of the urea cycle in linking N and C metabolism in diatoms [24], how OA would affect this process has not been documented yet. It is worth noting that RbcL protein levels have been shown to decrease in *T*. *weissflogii* and *E*. *huxleyi* [48], to increase in *T*. *pseudonana* (coastal strain) and *E*. *huxleyi* [40] or to be unaffected in *Trichodesmium* IMS101 [49] and *T*. *pseudonana* (offshore strain) [40], when grown under projected future CO_2_ levels, but in this study, both the down and up-regulation of the *rbcl* gene under the OA conditions were observed, showing the multifarious and species-specific nature of the effects.

In diatoms, the fucoxanthin-chlorophyll protein (FCP) is bound to chlorophylls *a*, *c* and the carotenoid fucoxanthin (Fuco), as the major complex in light-harvesting centers. About six FCP genes have been reported in *P*. *tricornutum* [50], and are known to show differential responses when exposed to different light levels [51]. In the present study, although the genes encoding FCP and mtATP synthase were slightly suppressed during the initial phase of OA (at the initial 1 day), they increased their expression levels under the OA treatment after the cells had acclimated for 6 generations, indicating a homeostatic response to an acidic perturbation to the cells [52]. The diel changes in the gene expression of the Lhcf 3 protein, being the lowest in the dark and the highest at the late light period, reflects a light dependency of this gene.

In brief, the physiological responses of the diatom *P*. *tricornutum* to OA were found to be linked with Ci acquisition, nitrite reduction, respiration and photosynthetic and photoprotective processes, with expression of the related genes up- or down-regulated. This work is the first attempt to elucidate the time-dependence of molecular responses underlying observed physiological changes in diatoms grown under OA, which could be mediated by the light/dark periods and circadian rhythms. On the other hand, adaptation to OA over a thousand generations showed decreased growth and cell size in the diatom *P*. *tricornutum* [53], implying a possibility that changes in molecular responses from acclimation to adaptation could have occurred, resulting in differential physiological performance. This requires further investigation in the future.

## Supporting information

S1 Fig The concept map of the experiment design. (DOCX)

S1 Table Chemical parameters of the seawater carbonate system. (DOCX)

S2 Table Nucleotide sequences of primers. (DOCX)
