# Supplementary figures and images for: Ocean acidification modulates expression of genes and physiological performance of a marine diatom
Source: PLoS One. 2017 Feb 13;12(2):e0170970. doi: 10.1371/journal.pone.0170970 (PMC5305191; doi:10.1371/journal.pone.0170970)

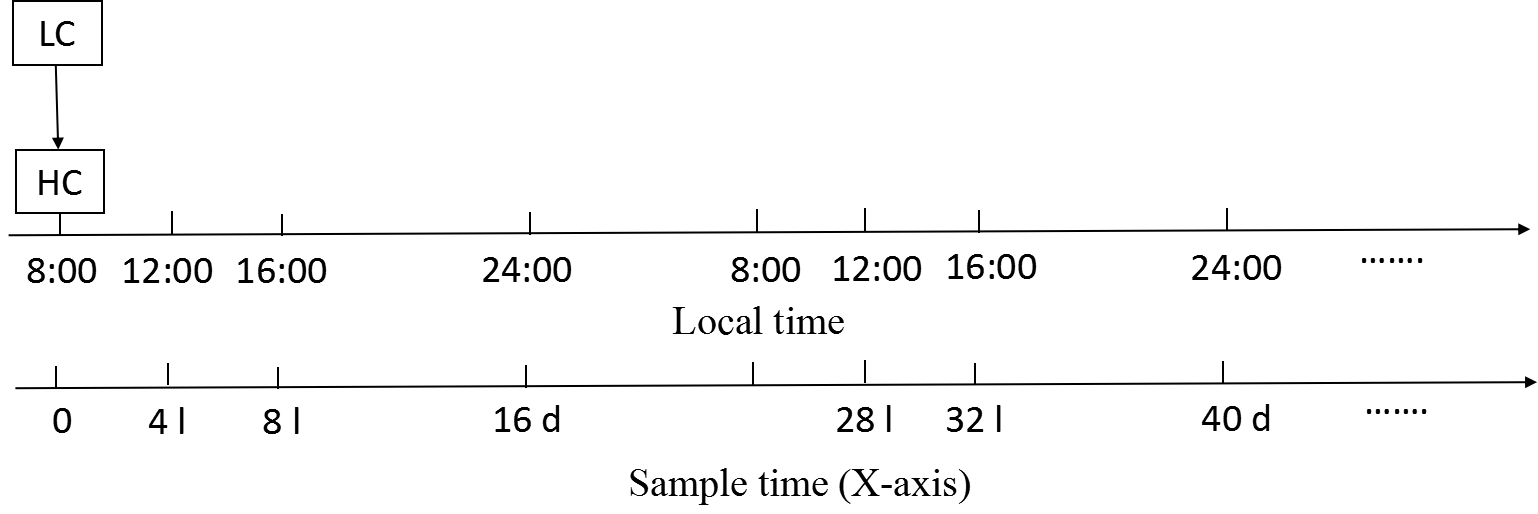


S1 Fig. The concept map of the experiment design.

Supplement: S1 Fig — (DOCX) [file pone.0170970.s001.docx]
