# Supplementary material for: Ocean acidification modulates expression of genes and physiological performance of a marine diatom
Source: PLoS One. 2017 Feb 13;12(2):e0170970. doi: 10.1371/journal.pone.0170970 (PMC5305191; doi:10.1371/journal.pone.0170970)
Supplement: S1 Table — (DOCX) [file pone.0170970.s002.docx]

**S1 Table** Chemical parameters of the seawater carbonate system. Carbonate chemistry parameters of the growth medium for ambient (390 μatm; LC) and elevated CO_2_ (1000 μatm; HC) cultures. TA stands for total alkalinity. The values are means ± SD, n = 3. Different superscripted letters represent significant difference between the ambient and acidified conditions.

| pCO_2_ | pH_NBS_ | DIC  (μmol kg^-1^) | HCO_3_^-^  (μmol kg^-1^) | CO_3_^2-^  (μmol kg^-1^) | CO_2_  (μmol kg^-1^) | Total alkalinity  (μmol kg^-1^) |
| --- | --- | --- | --- | --- | --- | --- |
| LC | 8.19±0.02^a^ | 2025.4±85.8^a^ | 1809.5±70.0^a^ | 203.3±15.6^a^ | 12.6^a^ | 2319.3±104.1^a^ |
| HC | 7.83±0.02^b^ | 2208.9±92.6^b^ | 2072.5±84.2^b^ | 104.0±8.4^b^ | 32.3^b^ | 2336.9±102.8^a^ |

The parameters of the seawater carbonate system (Table S1) were calculated from pH and pCO_2_ measurements with CO2 SYS software (Lewis and Wallace, 1998), using the values for the equilibrium constants K_1_ and K_2_ for carbonic acid dissociation from Roy et al. (1993) and that for K_B_ for boric acid of Dickson (1990). The pH change was determined with a pH meter (pH510, OAKTON) which was calibrated with standard National Bureau of Standards (NBS) buffer solutions (Hanna).

**Reference**

Dickson AG. Standard potential of the reaction: AgCl (s) + ^1^/_2_ H_2_ (g) = Ag (s) + HCl (aq), and the standard acidity constant of the ion HSO_4_ˉ in synthetic seawater from 273.15 to 318.15 K. J. Chem. Thermodyn. 1990; 22: 113-127.

Lewis E, and Wallace DWR. Program Developed for CO_2_ System Calculations, ORNL/CDIAC-105, Carbon Dioxide Information Analysis Center, Oak Ridge National Laboratory, US Department of Energy, 1998.

Roy RN, Roy LN, Vogel KM, Porter-Moore C, Pearson T, Good CE, Millero FJ, and Campbell DM. The dissociation constants of carbonic acid in seawater at salinities 5 to 45 and temperature 0 to 45ºC, Mar. Chem., 1993; 44: 249-267.
