# Supplementary material for: Ocean acidification modulates expression of genes and physiological performance of a marine diatom
Source: PLoS One. 2017 Feb 13;12(2):e0170970. doi: 10.1371/journal.pone.0170970 (PMC5305191; doi:10.1371/journal.pone.0170970)
Supplement: S2 Table — (DOCX) [file pone.0170970.s003.docx]

**S2** **Table** Nucleotide sequences of primers. Nucleotide sequences of primers used in the real-time quantitative PCR

| Gene | Primer name | Sequences(5’-3’) | Amplicon size (bp) |
| --- | --- | --- | --- |
| Histone H4 | H4-F | AGGCAAAGCGTGGTGTTCTTA | 156 |
|  | H4-R | TCTGGGGAGCCTCAGTCAATA |  |
| Synthase of mitochondrial ATP synthase | SM-F | AGGACAATACCAGCCCTACGAACCG | 147 |
|  | SM-R | ACCTTGGAGTGGACACCCTTGACAT |  |
| Nitrite reductase | NR-F | ATTGGGTGATTTCGCTTGAGAG | 182 |
|  | NR-R | CACCTCACTCGTCCCTTGTTCT |  |
| Fucoxanthin chlorophyll *a*/*c* protein, lhcf type | FC-F | CGGCTGGGACACCTTTGACG | 197 |
|  | FC-R | ATCTTGGAAACGACGGCAGTATC |  |
| Carbonic anhydrase | CA-F | TGGGAACTGAGGCTGGAACC | 162 |
|  | CA-R | AAGCACGGACACCACCACATT |  |
| NADH dehydrogenase subunit2 | NADH-F | TATTGGTTGCGGTGTTAGGTC | 155 |
|  | NADH-R | GAAATACTTAATACCCGCCTCA |  |
| Peroxisomal membrane protein-related | PMP-F | ATCTTGGTGGTGTAATCGTCC | 205 |
|  | PMP-R | GTTCCTTTGGTTTCCTCCTG |  |
| Ribulose-1,5-bisphosphate carboxylase/oxygenase large subunit | Rbcl-F | TCAATACTTCGCTTTTATCGCAT | 176 |
|  | Rbcl-R | CAGTAGCAGGACCTTGGAACG |  |
